# Adsorption behavior of biochar pyrolyzed from *barracuda grass* for cadmium ions

**DOI:** 10.3389/fchem.2022.971540

**Published:** 2022-08-17

**Authors:** Yan Shao, Zhiliang Chen, Zhonglei Zhang, Jun Pang, Yinyin Li, Jia Zhu, Gen Zhang, Xiaoshu Wang, Ming Chang, Lei Wang

**Affiliations:** ^1^ Institute for Thermal Power Engineering, State Key Laboratory of Clean Energy Utilization, Zhejiang University, Hangzhou, China; ^2^ Wuhan City Environment Protection Engineering Limited Company, Wuhan, China; ^3^ Guangdong Engineering Technology Research Center of Heavy Metal Pollution Control and Restoration in Farmland Soil, South China Institute of Environmental Sciences, Ministry of Ecology and Environment, Guangzhou, China; ^4^ China North Energy Conservation and Environment Protection Co, Ltd, Beijing, China; ^5^ College of Arts, Shandong Agricultural University, Taian, China; ^6^ School of Materials and Environmental Engineering, Institute of Urban Ecology and Environment Technology, Shenzhen Polytechnic, Shenzhen, China; ^7^ School of Ecology and Environment, Anhui Normal University, Wuhu, China; ^8^ State Key Laboratory of Environmental Criteria and Risk Assessment, Chinese Research Academy of Environmental Sciences, Beijing, China

**Keywords:** barracuda grass biochar, adsorption model, cadmium, soluble cations, mechanism

## Abstract

This work investigated the removal of cadmium ions (Cd^2+^) by using biochar derived from *Barracuda Grass*. The biochars derived from the pyrolysis of roots (BGR), stems (BGS) and leaves (BGL) were characterized and their performance for cadmium adsorption was studied at varying parameters of temperature, time, and alkali (earth) metal concentrations. The maximum adsorption amount at equilibrium of BGR, BGS and BGL was determined experimentally as 8.38 mg/g, 42.12 mg/g, and 30.39 mg/g. Adsorption fitting results revealed that Cd2+ adsorbed on BGR and BGS preferred to be multilayer-covered, and BGL was more likely to have monolayer-covered functions. The kinetic data fitted well to the pseudo-second-order model (*R* ^2^ > 0.99), revealing the adsorption process was a spontaneous monolayer chemisorption process. The results of alkaline (earth) metals leaching of biochar revealed that the inherent alkaline (earth) metals in biochar made inhibitory functions on the Cd^2+^ adsorption behavior by occupying the active sites. And in the process of wastewater treatment, the leaching of alkaline earth metals might enhance the complexation reaction between surface groups and Cd^2+^. This study provides a feasible strategy for the resource utilization of abundant hydrophytic plants in waste management.

## 1 Introduction

A huge number of cadmium (Cd) ions have been released into the environment by widespread industries such as electric batteries, electronic components, pigments, and fertilizers (Wang et al., 2015; Shi et al., 2019), causing pollution and garnering worldwide concern. Once entering the aquatic ecosystem, cadmium inhibits plant growth by affecting photosynthesis and enzyme activities, and finally damages the key organs of animal organism and restrains metabolism. According to statistics, the farmland field with Cd pollution has exceeded 20×10^4^ hm^2^ (Wang et al., 2021). Adsorption has been proved a cost-effective technology to duce the toxicity and chemical activity of Cd pollution in the media, with the advantages of being widely available, high affinity, simple design, and easy operation (Che et al., 2020). Since the application of the adsorption strategy is highly dependent on the performance of adsorbents, it is of great importance to developing an environmental-friendly, easy-available and low-cost Cd ions adsorbent.

Recently, biochar-based material has attracted a lot of attention for its efficient application in carbon sequestration and water pollution control (Zou et al., 2016). Especially, biochar shows great affinity for heavy metals due to the wide source of raw material, low cost, high porosity, large specific surface area and abundant surface functional group (Zhang et al., 2014). However, the adsorption capacity of biochar is still limited by raw materials, experimental conditions and co-existing ions. Studies (Don et al., 2022; Wentao et al., 2022) showed that pyrolysis process under ambient conditions made it possible to convert waste biomass into value-added biochar in dealing with heavy metals, such as coconut shell biochar (Cr-99.9%), rape straw biochar (Pb-100%), perilla leaf biochar (As-100%), and enteromorpha prolifera biochar (Cd-99.8%), and affected by the co-existing ions significantly (Luo et al., 2019).

Although there have been studies on Cd ions removal by biochar adsorption, the effects of different plant organs on the adsorption capacity of biochar have rarely been reported, which limits the practical application of biochar in the field of Cd ions removal. In this work, biochars derived from the pyrolysis of root (BGR), stem (BGS), and leaf (BGL) of *Barracuda Grass* were studied as adsorbents for the removal of Cd^2+^ respectively. The physical structures and surface chemical properties of the biochar were characterized. The adsorption mechanism of Cd^2+^ was explored by isothermal and kinetic models, and the leaching performance of alkali (earth) metals was also investigated. Overall, this study provides a reference for the preparation of biochar from plant roots, stems, and leaves for remediation of heavy metal pollution.

## 2 Materials and methods

### 2.1 Biochar preparation and characterization

BGR, BGS, and BGL originated from the pyrolysis of roots, stems and leaves of *Barracuda Grass* respectively. In each pyrolysis treatment, 5.0 g biomass was heated to 600°C with a heating rate of 5°C/min and then kept for 2 h in flowing N_2_. The biochar derived from pyrolysis was then rinsed with deionized water to the neutral and dried at 60°C. Finally, the obtained biochar was crushed and sieved to particle size less than 100 mesh (0.150 mm) and prepared for further experiments.

The Physical and chemical properties of biochar samples were characterized. The specific surface area and pore structure were characterized by the Brunauer–Emmett–Teller N_2_ adsorption method. The surface morphologies were determined by scanning electron microscopy (SEM). The crystalline properties were observed with an X-ray diffractometer (XRD). The functional groups on the surface of biochar were analyzed by Fourier transform infrared spectrometer (FTIR). Besides, the metal ion concentration was obtained by an inductively coupled plasma optical emission spectrometer (ICP-OES).

### 2.2 Batch experiments

For all prepared synthetic solutions, NaNO_3_ (0.01 mol/L) was used as the supporting electrolyte in order to simulate the conditions of real wastewater as much as possible. Certain concentrations of Cd^2+^ (5–30 mg/L) were prepared and pH value was adjusted to 5.5 with HNO_3_ and NaOH, which was used to evade the precipitation effect without affecting the study of the adsorption process (Jiang et al., 2020). 0.02 g biochar was accurately weighed and bottled into a 50 ml polyethylene centrifuge tube. Subsequently, 20 ml of stock solution was added and shaken in an oscillating chamber at the given temperature (288.15K–318.15 K) for 1–1440min and passed through 0.22 μm aqueous filter membranes. Then, the supernatant was examined by ICP-OES to determine the concentration of metal ions (Cd^2+^, K^+^, Ca^2+^, and Mg^2+^). Once obtained Cd^2+^ concentration, the equilibrium adsorption quantity *q*_*e*_ (mg/g) and the removal efficiency (*RE*, %) were calculated according to the following formulae:

$$q_{e}\mathrm{=(}C_{0}-C_{e}\mathrm{)V/m}$$ (1)

$$RE=\frac{C_{0}-C_{e}}{C_{0}}\times 100\%$$ (2)

where *C*_*0*_ and *C*_*e*_ are the Cd^2+^ concentrations at the initial and equilibrium condition (mg/L), *V* is the volume of solution (L), and *m* is the mass of biochar (g).

### 2.3 Adsorption models

Adsorption isotherms and kinetics models can reveal important information about adsorption behavior (Zhang et al., 2015). Here, Langmuir and Freundlich models were used as isotherms to fit the experimental data, and kinetic curves were fitted with pseudo-first-order, pseudo-second-order, and intra-particle diffusion models, which are as follows:

$$q_{e}=\frac{k_{l}q_{m}C_{e}}{1+k_{l}C_{e}}$$ ( 3)

$$q_{e}=k_{f}C_{e}^{\frac{1}{n}}$$ (4)

$$\mathrm{ln(}q_{e}-q_{t}\mathrm{)=ln}q_{e}-k_{1}t$$ (5)

$$\frac{t}{q_{t}}=\frac{1}{k_{2}q_{e}^{2}}+\frac{t}{q_{e}}$$ (6)

$$q_{t}=k_{p}\mathrm{t+C}$$ (7)

where, *q*_*m*_, *q*_*t*_ and *q*_*e*_ are the adsorption capacities at maximum, time t and equilibrium, mg/g; *k*_*l*_ is the Langmuir isothermal parameter, L/g; *k*_*f*_ is the adsorption capacity of Freundlich equation, mg/g; *n* is the adsorption intensity. *k*_*1*_, *k*_*2*_ and *k*_*p*_ are the rate constants of pseudo-first-order kinetic/min, pseudo-second-order kinetic (g/(mg·min)), and intra-particle diffusion model (mg/(min^1/2^·g)), and *C* is the intercept, mg/g.

## 3 Results and discussion

### 3.1 Characterization

**FIGURE 1:**
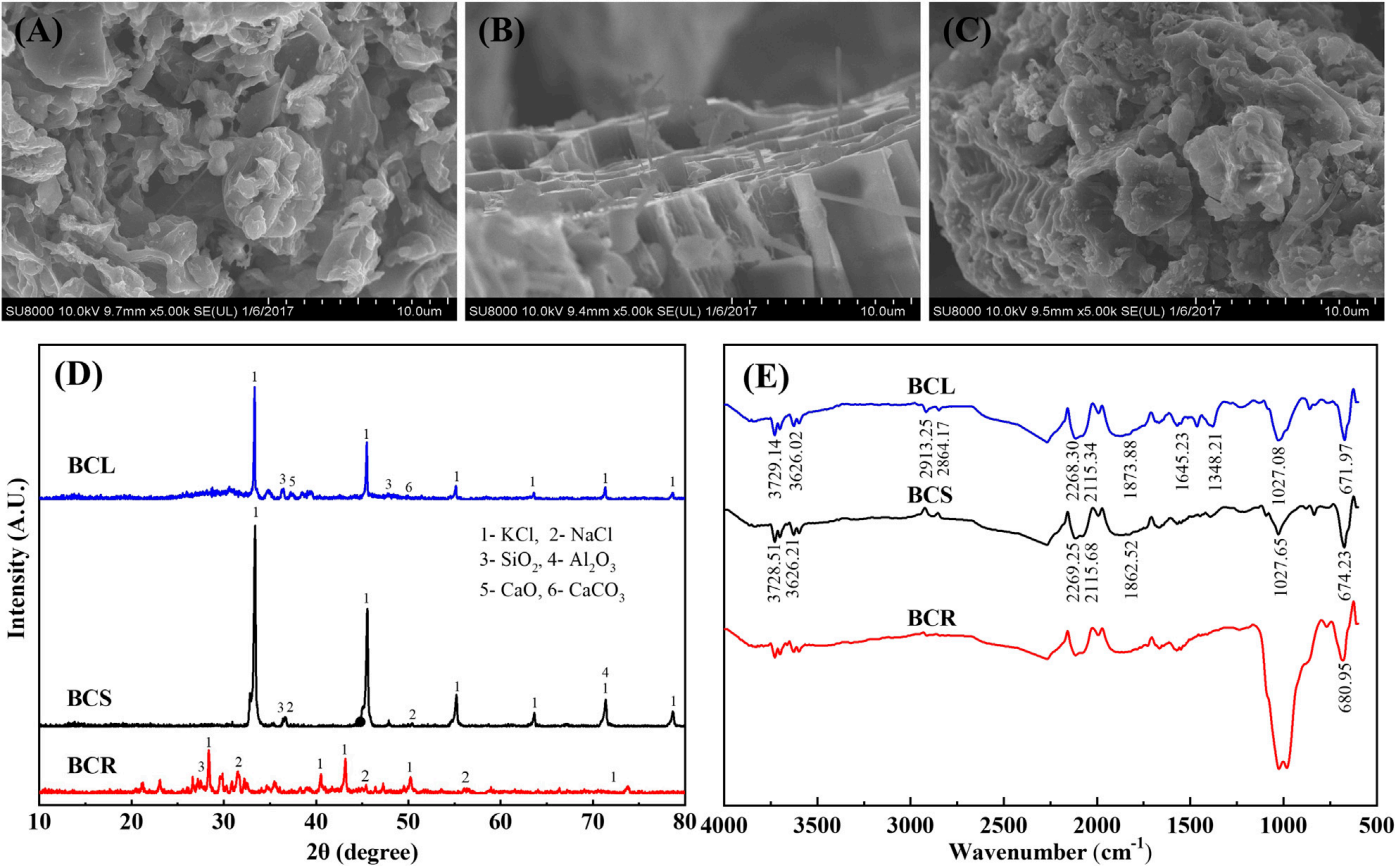
SEM graphs of **(A)** BGR, **(B)** BGS, **(C)** BGL; **(D)** XRD patterns of the BGR, BGS, BGL; **(E)** FT-IR spectrum of the BGR, BGS, BGL.

The yields of BGR, BGS, and BGL in this study didn’t differ much, all-around 30%. The characterization results showed that the specific surface area of the BGS (317.24 m^2^/g) was far greater than that of BGR (49.15 m^2^/g) and BGL (5.60 m^2^/g). And the corresponding pore volumes of BGR, BGS and BGL were respectively 0.05 cm^3^/g, 0.17 cm^3^/g, and 0.01 cm^3^/g. The SEM result was shown in Figures 1A–C. The surface structures of BGR, BGS, and BGL were all loose and irregular, but the pore structure of BGS was developed better than that of BGR and BGL, which provides BGS larger surface area and more adsorption sites available for adsorbing Cd^2+^. XRD was employed to further confirm the phase composition of BGR, BGS, and BGL. As shown in Figure 1D, the main peaks of KCl and weak peaks of SiO_2_ appeared in all samples (Huang et al., 2020). Typically, plant-based biochar is rich in KCl components due to the fact that K is an essential nutrient for plant growth (Zhang et al., 2013). In contrast to BGS and BGR, weakly intense peaks of CaO and CaCO_3_ were observed in BGL, indicating the presence of more mineral phases in BGL.

Figure 1E showed the typical FT-IR spectrum ranged between 4000cm^−1^ and 400cm^−1^ of BGR, BGS, and BGL. The broad peak at 680cm^−1^-1027cm^−1^ was observed in all the samples, indicating C-O stretching vibrations and O-H out-of-plane bending were present in BGR, BGS, and BGL (Ozciimen and Mericboyu, 2010). The peaks observed at 2913cm^−1^, and 2864cm^−1^ could be assigned to the C-H stretching vibration of aromatic compounds (Chen et al., 2008), indicating the functional groups present on the biochar may be responsible for Cd(II) sorption. The broad peaks at 3728cm^−1^, 3626cm^−1^, and 1870cm^−1^ could be assigned to O-H and C=O stretching vibration (Atkinson et al., 2010), revealing the existence of oxygen-containing functional groups such as carboxyl, carbonyl, and ester groups, which could provide adsorption sites for biochar adsorption by ligand complexation with heavy metals (Pyrzyńsk and Bystrzejewski, 2010; Yang et al., 2019). In addition, the broad peaks at 1616cm^−1^ attributed to C=C stretching vibration were only observed in BGS, implying the feature of unsaturated olefins (He et al., 2019). Meanwhile, the signals at 1463cm^−1^ and 1379cm^−1^ were only observed in BGL, corresponding to the stretching mode of C-H bending vibration and CH_3_ symmetric angle change. The functional groups on the surface of biochar can provide active sites for the immobilization of heavy metals by complexation, contributing to the adsorption of Cd^2+^ from water bodies (Tan et al., 2015).

### 3.2 The effect of temperature on Cd^2+^ adsorption

**FIGURE 2:**
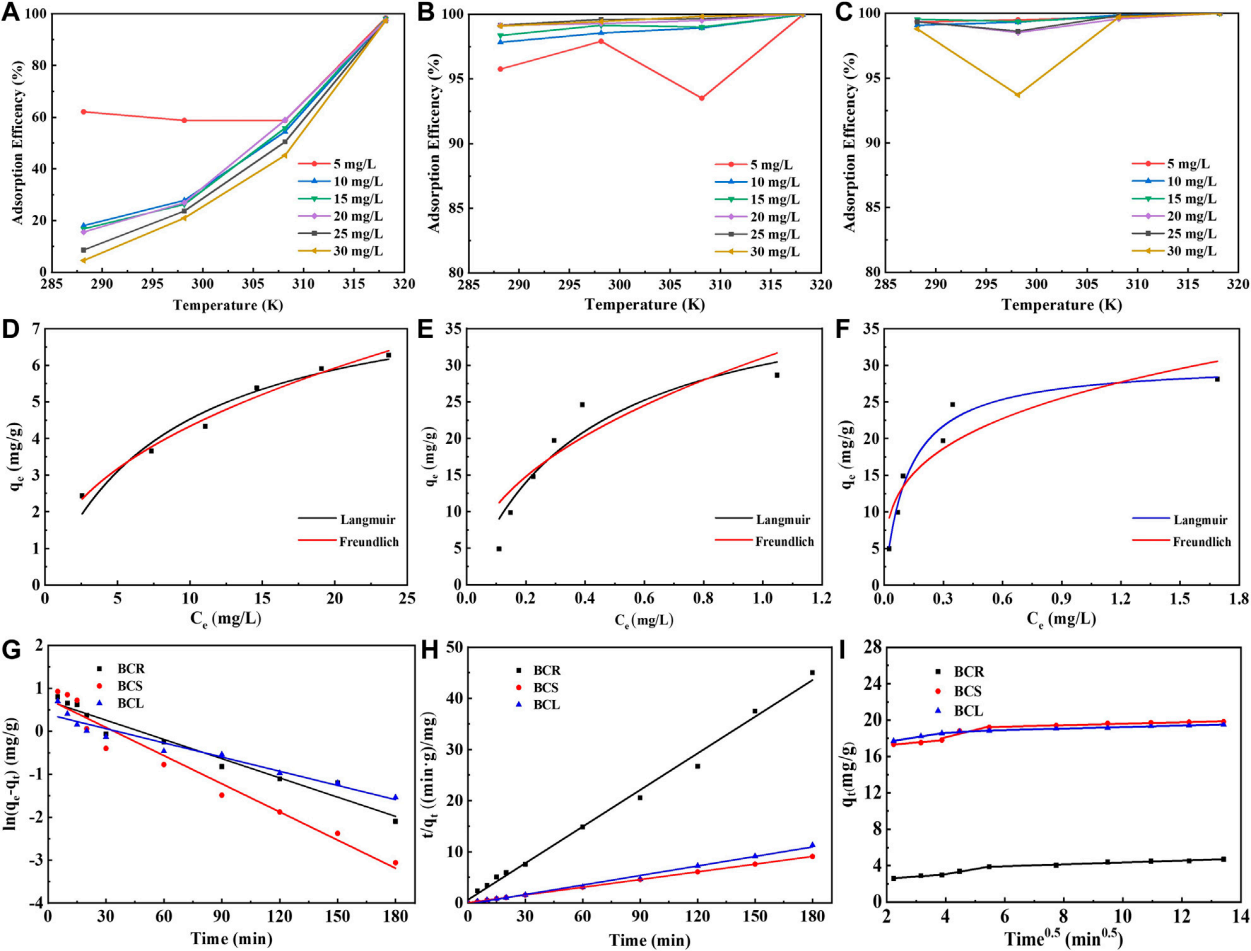
Effect of temperature on Cd^2+^ adsorption by **(A)** BGR, **(B)**BGS, **(C)**BGL; Isotherms of Cd^2+^ adsorption by **(D)** BGR, **(E)**BGS, **(F)**BGL; Kinetic fittings of Cd^2+^ adsorption by **(G)** Pseudo-first-order, **(F)** Pseudo-second-order, and **(I)** Intra particle diffusion.

For sustainable industrial wastewater treatment, it is necessary to figure out the effects of different operating parameters on Cd^2+^ adsorption, such as the temperature (Xiong et al., 2017). The adsorption experiments of Cd^2+^ with BGR, BGS, and BGL at different temperatures were conducted and the results were shown in Figures 2A–C. With the increasing temperature from 288.15K to 318.15K, the adsorption efficiency of BGR showed an upward trend and eventually reached 97–98%. It is could be ascribed to the mobility of adsorbed ions increasing with the increase of temperature, thus increasing the adsorption capacity of the BGR. The effects of temperature on the adsorption efficiency of BGS and BGL to Cd^2+^ were basically similar. When the temperature increased, the adsorption efficiency of BGS and BGL kept at a high level and didn’t change much. The plant roots (BCR) are always submerged in a constant temperature water environment for growth. When the external temperature changes significantly, the surface group distribution and structure change accordingly.

### 3.3 Model analysis

The fitting results of the isotherm models were shown in Figures 2D–F. The maximum adsorption amount at the equilibrium of BGR, BGS, and BGL was determined experimentally as 8.38 mg/g, 42.12 mg/g, and 30.39 mg/g. The difference in adsorption capacity could be related to the number of active sites, specific surface area, and ionic affinity. As shown in Fig (d–f), for BGL, Langmuir model (*R* ^2^ = 0.9622) fitted better than Freundlich model (*R* ^2^ = 0.9622), indicating that Cd^2+^ adsorbed on BGL preferred to be monolayer-covered (Khan et al., 2019). For BGR and BGS, Freundlich model (*R* ^2^, BGR = 0.9832, BGS = 0.9820) fitted better than Langmuir model (*R* ^2^, BGR = 0.9427, BGS = 0.8730), indicating that Cd^2+^ adsorbed on BGR and BGS preferred to be multilayer -covered. All the biochar samples have a good affinity towards Cd^2+^since n < 1. Besides, the magnitude of KF also showed higher uptake of Cd^2+^ by BGS and BGL than that by BGR (Li et al., 2014), indicating that BGS and BGL could serve as better environmental sorbents to treat Cd^2+^in wastewater. Compared with many corresponding materials, *Barracuda Grass*-derived biochar showed some adsorption potential, especially for BGS and BGL (Wang et al., 2019). Of course, some modified biochar performed better than the adsorbents in this study. However, in order to improve their absorptive capacities, the modification required relatively many preparation steps, energy utilization, and higher material costs, which have no advantages in terms of cost economy.

To evaluate the adsorption characteristics, the pseudo-first-order, pseudo-second-order, and intra-particle diffusion modeled and experimentally measured values are presented in Table 1. The linear fit plots are shown in Figures 2G–I. The applicability of the three models indicated that the pseudo-second-order model (*R* ^2^ > 0.99) appeared to be the most accurate model to describe Cd^2+^ adsorption, suggesting the rate of adsorption was majorly governed by the chemisorption mechanism. The kinetic behavior of the microporous adsorbent indicates that the adsorption process may involve the internal surface diffusion of the particles. In order to better understand the sorption mechanism, the intra-particle diffusion model was used to analyze. As shown in Figure 2I, it was divided into the boundary layer of the surface, particle diffusion, and final equilibrium state, representing three stages of intra-particle diffusion process. The C_1_ value provides information about the boundary layer thickness in the adsorbent (BGS > BGL > BGR). The adsorption rate at the second stage is much higher than that at the first and third stages, which could be attributed to that Cd^2+^ might easily access the outer surface adsorption sites of the biochar samples through physical interactions, such as electrostatic attraction (Dumitru and Bulgariu, 2016). Obviously, the fitting curve did not pass through the origin point, suggesting that intra-particle diffusion was not exactly the only rate-limiting process (Yu et al., 2021).

**TABLE 1:** The pseudo-first-order, pseudo-second-order and intra particle diffusion model parameters for Cd^2+^ adsorption by biochar.

Samples	Parameters		BGR	BGS	BGL
Experimental	q_max_ (mg/g)		4.820	19.865	19.721
The pseudo-first-order model	q_e_ (mg/g)		2.026	2.104	1.479
	k_1_×10^–2^ (min^−1^)		1.493	2.185	1.102
	*R* ^2^		0.957	0.954	0.937
The pseudo-second-order model	q_e_ (mg/g)		4.190	19.904	16.132
	k_2_×10^–2^ (g/(mg·min))		9.300	4.665	1.851
	*R* ^2^		0.993	0.999	0.994
Intra-particle diffusion model	Step 1	C_1_ (mg/g)	2.109	16.682	16.557
		(k_p_)_1_ (mg/(min^1/2^·g))	0.229	0.283	0.519
		(*R* ^2^)_1_	0.831	0.921	0.995
	Step 2	C_2_ (mg/g)	0.791	14.849	17.884
		(k_p_)_2_ (mg/(min^1/2^·g))	0.567	0.815	0.178
		(*R* ^2^)_2_	0.987	0.707	0.929
	Step 3	C_3_ (mg/g)	3.309	18.794	18.408
		(k_p_)_3_ (mg/(min^1/2^·g))	0.104	0.0805	0.0826
		(*R* ^2^)_3_	0.952	0.945	0.975

On the whole, the pseudo-second-order model is perfectly consistent with the entire adsorption process of BGR, BGS, and BGL. These kinetic results revealed the adsorption depended on the chemisorption rate controlling mechanism (Liang et al., 2017). Besides, there are various velocity control factors for intra-particle diffusion, especially in the process of diffusion from the liquid phase boundary layer around the particle to the particle surface.

### 3.4 The effect of alkali (earth) metal concentration on Cd^2+^ adsorption

**FIGURE 3:**
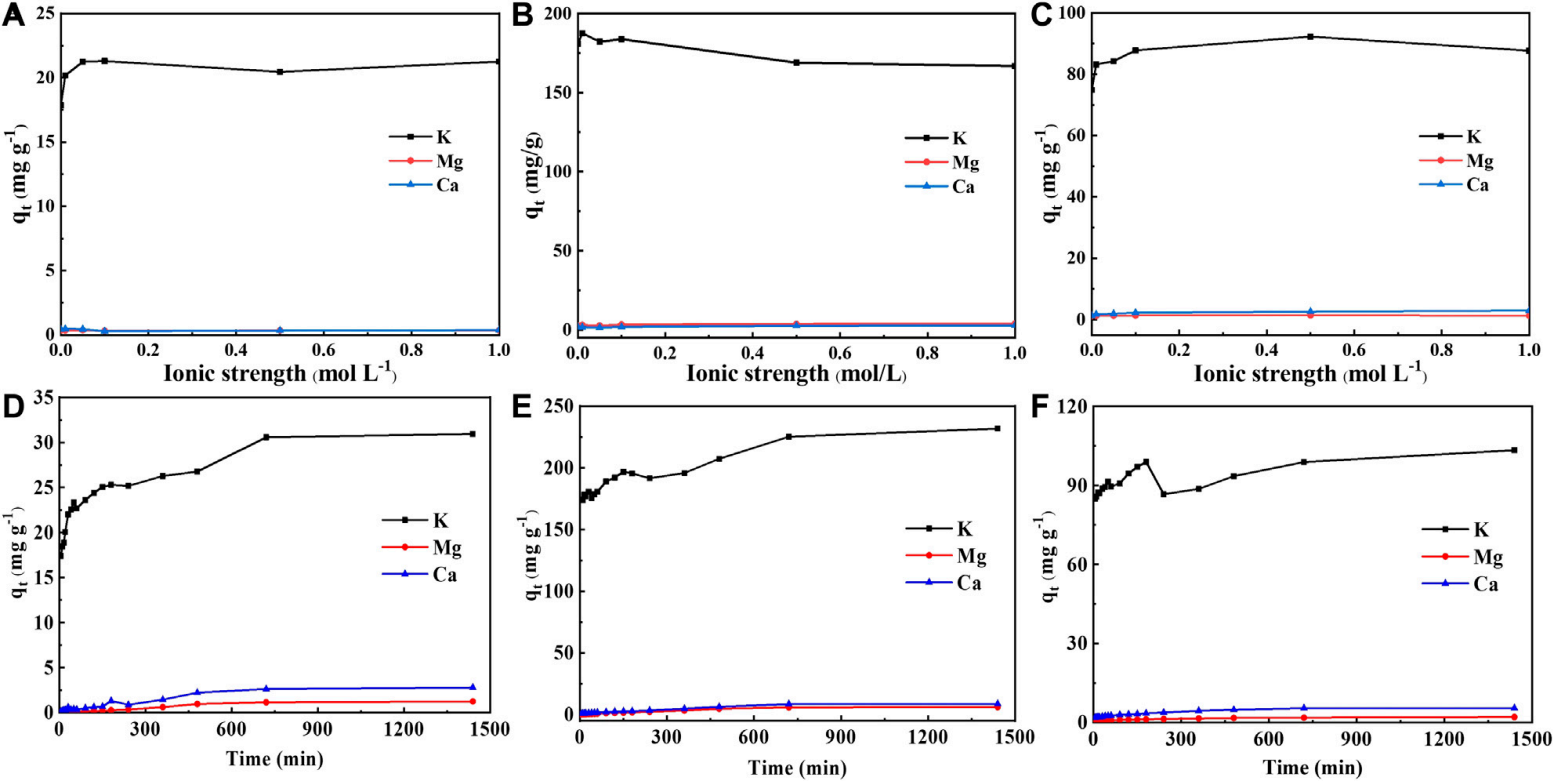
Effect of ionic strength on the leaching amount of inorganic components: **(A)** BGR, **(B)** BGS and **(C)** BGL; The amount of inorganic component leaching changes with time: **(D)** BGR; **(E)** BGS and **(F)** BGL.

Biomass absorbs a large amount of alkali and alkaline earth metals (mainly K, Ca, and Mg) as business elements during the growth process, thus their role in the adsorption process should not be overlooked. Figure 3 showed the effect of ionic strength (NaNO_3_) and time on the leaching amount of alkali (earth) metals. With the increase of ionic strength, the leaching amount of K^+^ increased firstly and then declined, while that of Mg^2+^ and Ca^2+^ remained basically unchanged. The leaching amount of K^+^ leaching amount reached up to 187 mg/g, which was ten times more than that of Mg^2+^ and Ca^2+^. When the ionic strength increased, the leaching amount of Ca^2+^ kept at around 3 mg/g for BGS and BGL, while the Ca^2+^ leaching amount in BGR ranged from 0.3 to 0.5 mg/g.

As shown in Figures 3D–F, the time evolutions of K^+^ leaching in BGR and BGS were similar. As time went on, the leaching amount of K^+^ in BGR and BGS slowly increased and then kept stable. In contrast, the leaching amount of K^+^ in BGL first rose sharply, then continued to rise after a steep decline, and finally became relatively stable. In addition, the leaching of K^+^ is much higher than that of Mg^2+^ and Ca^2+^ all the time, which is related to the intrinsic characteristics of aquatic plants (AhmedAlamin and Kaewsichan, 2015). The maximum K^+^ leaching in BGS reached 231 mg/g, followed by the maximum leaching of 103 mg/g in BGL and the lowest maximum leaching value of 30 mg/g in BGR. Due to the larger specific surface area of BGS, the inorganic fraction of which can provide more adsorption sites, the release of alkaline earth metals is significantly better than the remaining biochar and is beneficial to the adsorption of heavy metals.

Leaching experiments of alkali and alkaline earth metallic ions implied that the K^+^ in biochar mainly gave rise to Cd ions adsorption through ion exchange. Numerous ions still remained in biochar during the adsorption process, especially for Ca and Mg, which were probably forming crystals based on carbon matrix. Moreover, surface functional groups in biochar were assigned to have the affinity toward Cd ions through complexation during the adsorption process. This phenomenon was confirmed in C=C vibration signals at 1645cm^−1^ and 1348cm^−1^ and C=O stretching vibration at 1870cm^−1^ (Figure 1E).

## 4 Conclusion

In this paper, the characterization and Cd^2+^ adsorption behavior of *Barracuda Grass*-based biochar (BGR, BGS, and BGL) were investigated. The adsorption capability of biochar samples for removing Cd^2+^ was influenced by the contact conditions such as initial concentration, contact time, and temperature. The maximum adsorption amount at the equilibrium of BGR, BGS, and BGL was determined experimentally as 8.38 mg/g, 42.12 mg/g, and 30.39 mg/g, showing comparatively good adsorption performance. Adsorption fitting results revealed that Cd^2+^ adsorbed on BGR and BGS preferred to be multilayer-covered, and BGL was more likely to have monolayer-covered functions. Kinetics studies further reflected the adsorption behavior of the three biochar could be well fitted by the pseudo-second-order model, suggesting the process was heterogeneously chemical adsorption. With the increase of ionic strength, the leaching amount of K^+^ increased firstly and then declined, while that of Mg^2+^ and Ca^2+^ remained basically unchanged. The adsorption of Cd^2+^ by BGR, BGS, and BGL was dominated by ion exchanging with K^+^ and complexing with C=O and C=C. The leaching of alkaline earth metals might be beneficial to removing Cd^2+^, thus favoring the use of plant-based biochar in the treatment of wastewater-contaminated heavy metals.

## Data Availability

The original contributions presented in the study are included in the article/supplementary material, further inquiries can be directed to the corresponding authors.
